# Supplementary material for: Characterizing partisan political narrative frameworks about COVID-19 on Twitter
Source: EPJ Data Sci. 2021 Oct 30;10(1):53. doi: 10.1140/epjds/s13688-021-00308-4 (PMC8556838; doi:10.1140/epjds/s13688-021-00308-4)
Supplement: Supplementary file 1 — Supplementary information (PDF 79 kB) [file 13688_2021_308_MOESM1_ESM.pdf]

Supplementary Material for: Characterizing Partisan Political Narrative Frameworks about COVID-19 on Twitter

August 26, 2021

Top Democratic tokens compared to backgound	Top Republican tokens compared to backgound	Common over-represented tokens between two parties
outbreak, novel, symptoms, hospitalized, related, vaccine, reessex, beshear, presumptive, teletown	novel, outbreak, msdh, wvdhhr, azdhs, hospitalized, recovered, plasma, hysteria, preparations	outbreak, novel, symptoms, hospitalized, presumptive, recovered, plasma, shortness' spreads, slowing

Table 1: Over-represented Democratic and Republican tokens compared to the background corpus, and the common over-represented tokens between two parties, computed by comparing the difference in the dense rank for each token in each corpus.

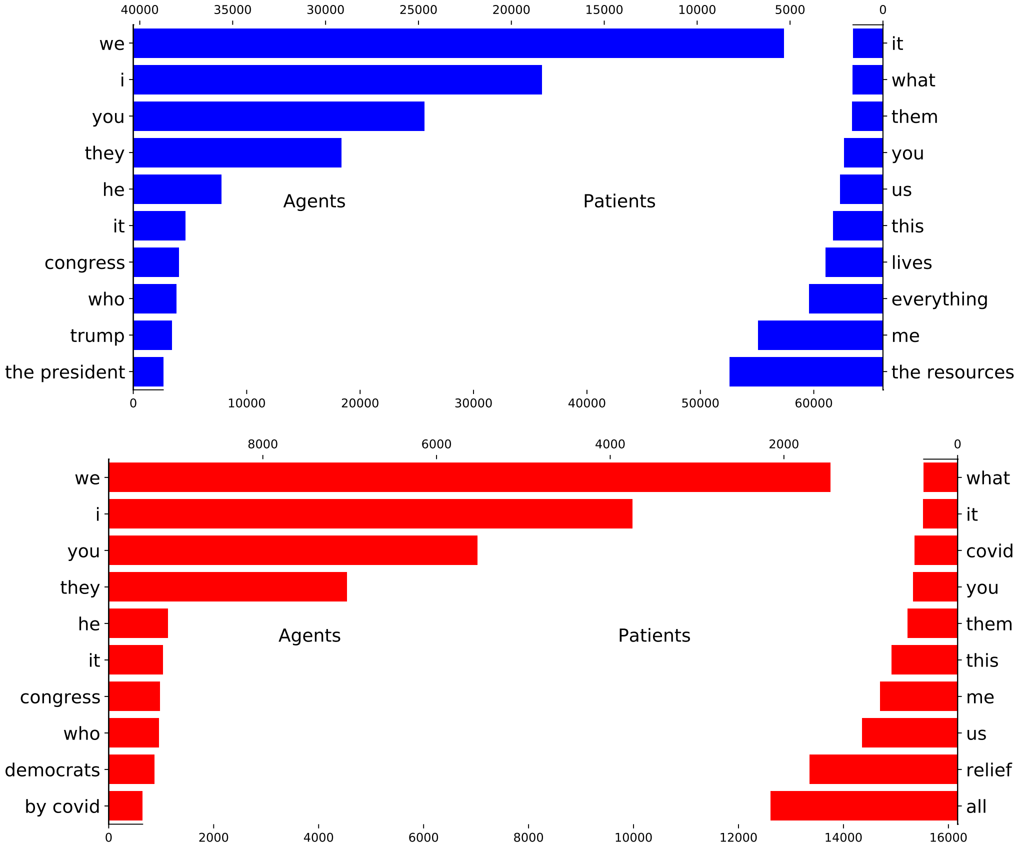

Figure 1: Ten most frequent Agents and Patients in Democratic and Republican tweets, with their frequencies. Top figure shows the Democratic Agents and Patients, and bottom figure shows the Republican ones.

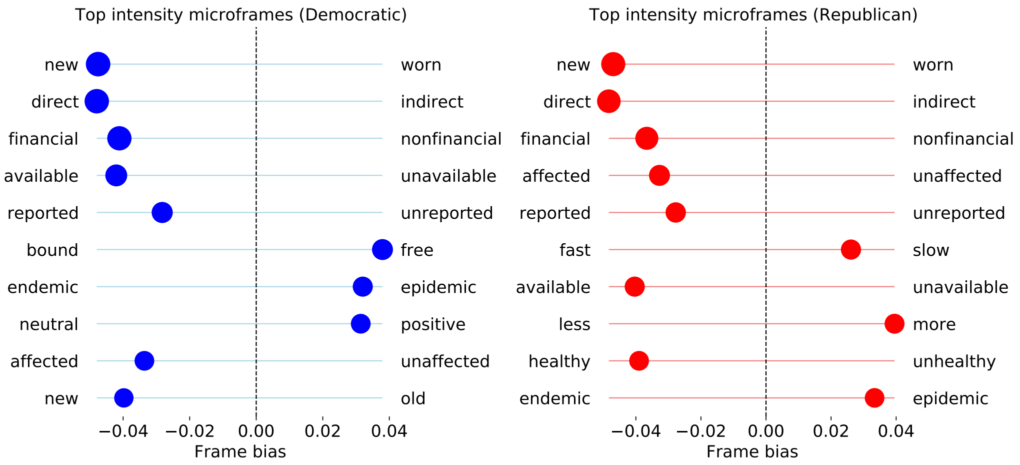

Figure 2: Top 10 microframes with highest intensity values for each party, as well as their frame bias. The position of points indicate the values of bias, and the size of points indicate the values of intensity. The tick labels are the poles of the microframes.
